# p38MAPK, ERK and PI3K Signaling Pathways Are Involved in C5a-Primed Neutrophils for ANCA-Mediated Activation

**DOI:** 10.1371/journal.pone.0038317

**Published:** 2012-05-31

**Authors:** Jian Hao, Li-Qiang Meng, Peng-Cheng Xu, Min Chen, Ming-Hui Zhao

**Affiliations:** 1 Renal Division, Key Laboratory of Renal Disease, Department of Medicine, Peking University First Hospital, Institute of Nephrology, Peking University, Ministry of Health of China, Beijing, China; 2 Renal Division, Department of Medicine, The Affiliated Hospital of Inner Mongolia Medical College, Huhehot, Inner Mongolia, China; Massachusetts General Hospital/Harvard Medical School, United States of America

## Abstract

**Background:**

The complement system is one of the important contributing factors in the development of antineutrophil cytoplasmic antibody (ANCA)-associated vasculitis (AAV). C5a and the neutrophil C5a receptor play a central role in antineutrophil cytoplasmic antibody (ANCA)-mediated neutrophil recruitment and activation. The current study further investigated the signaling pathways of C5a-mediated priming of human neutrophils for ANCA-induced neutrophil activation.

**Methodology/Principal Findings:**

The effects of the p38 mitogen-activated protein kinase (p38MAPK) inhibitor (SB202190), extracellular signal-regulated kinase (ERK) inhibitor (PD98059), c-Jun N-terminal kinase (JNK) inhibitor (6o) and phosphoinositol 3-kinase (PI3K) inhibitor (LY294002) were tested on respiratory burst and degranulation of C5a-primed neutrophils activated with ANCA, as well as on C5a-induced increase in expression of membrane-bound PR3 (mPR3) on neutrophils. For C5a-primed neutrophils for MPO-ANCA-induced respiratory burst, the mean fluorescence intensity (MFI) value was 254.8±67.1, which decreased to 203.6±60.3, 204.4±36.7, 202.4±49.9 and 188±47.9 upon pre-incubation with SB202190, PD98059, LY294002 and the mixture of above-mentioned three inhibitors (compared with that without inhibitors, *P*<0.01, *P*<0.05, *P<0.01* and *P*<0.05), respectively. For PR3-ANCA-positive IgG, the MFI value increased in C5a-primed neutrophils, which decreased upon pre-incubation with above-mentioned inhibitors. The lactoferrin concentration increased in C5a-primed neutrophils induced by MPO or PR3-ANCA-positive IgG supernatant and decreased upon pre-incubation with above-mentioned three inhibitors. mPR3 expression increased from 923.3±182.4 in untreated cells to 1278.3±299.3 after C5a treatment and decreased to 1069.9±188.9, 1100±238.2, 1092.3±231.8 and 1053.9±200.3 by SB202190, PD98059, LY294002 and the mixture of above-mentioned three inhibitors (compared with that without inhibitors, *P*<0.01, *P*<0.05, *P*<0.01 and *P*<0.01), respectively.

**Conclusions/Significance:**

Activation of p38MAPK, ERK and PI3K are important steps in the translocation of ANCA antigens and C5a-induced activation of neutrophils by ANCA.

## Introduction

Antineutrophil cytoplasmic antibody (ANCA)-associated vasculitis (AAV) comprises granulomatosis with polyangiitis (GPA, previously named Wegener's granulomatosis), microscopic polyangiitis (MPA) and Churg-Strauss syndrome (CSS). [1]. ANCAs are the serological hallmarks for the above-mentioned primary small vessel vasculitis. Proteinase 3 (PR3) and myeloperoxidase (MPO) are the two most important target antigens of ANCA in AAV.

Increasing evidences suggest that ANCA-induced neutrophil activation plays an important role in the pathogenesis of AAV. In vitro, ANCAs activate primed neutrophils to undergo a respiratory burst and degranulation, which may play a direct pathogenic role in vasculitic lesion development [2]–[6]. In an anti-MPO antibody-induced mouse vasculitis model [7], ANCA and neutrophils are necessary for the initiation of glomerulonephritis [7], [8]. Recent studies, both in the mouse model and in human, suggested that complement activation *via* the alternative pathway is one of the important contributing factors in the disease development [9]–[11]. Schreiber et al. further found that recombinant C5a dose-dependently primes neutrophils for ANCA-induced respiratory burst. As such, C5a and the neutrophil C5a receptor may compose an amplification loop and thus, plays a central role in ANCA-mediated neutrophil recruitment and activation [12]. However, little is known about the intracellular events that control ANCA-mediated activation of C5a-primed neutrophils.

Mitogen-activated protein kinases (MAPK) are activated via phosphorylation of threonine and tyrosine residues by upstream dual-specificity kinases and provide potent inflammatory signaling pathways [13], [14]. The p38MAPK and extracellular signal-regulated kinase (ERK), but not c-Jun N-terminal kinase (JNK), are responsible for the tumor necrosis factor-α (TNF-α)-primed neutrophils enabling subsequent ANCA-induced respiratory burst; however, only p38MAPK has been demonstrated to be responsible for translocation of ANCA antigens to the cell surface [15], [16]. Phosphoinositol 3-kinase (PI3K) signaling pathway controls various C5a-mediated effects on neutrophil and monocyte innate immunity and exerts an overall protective effect during experimental sepsis [17]. It has been reported that inhibition of phosphoinositol 3 kinase-γ isoform (PI3Kγ) protected the mouse from developing ANCA-associated necrotizing crescentic glomerulonephritis (NCGN). Inhibition of PI3Kγ blocks ANCA-induced Akt phosphorylation in TNFα-primed neutrophils [18]. Therefore, we hypothesized that the p38MAPK, ERK and PI3K might be involved in C5a-primed neutrophils for ANCA-mediated respiratory burst and degranulation.

**Figure 1:**
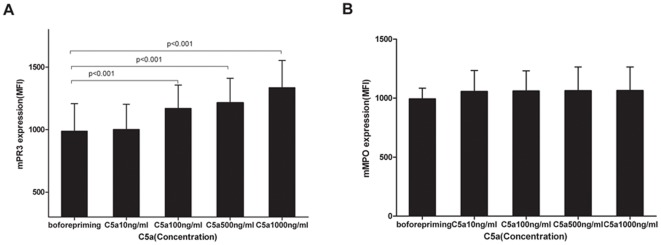
C5a increased membrane expression of PR3 (mPR3) on neutrophils. Human neutrophils were isolated and incubated with different concentrations of C5a for 15 min. mPR3 and mMPO were measured with flow cytometry and compared with non-stimulated cells. A: mPR3 expression compared with non-primed nertrophils (n = 11). B: mMPO expression compared with non-primed neutrophils (n = 11). Bars denoted means±SD of mPR3 or mMPO expression (MFI). Differences of MFI between groups were assessed using the t test.

## Materials and Methods

### Preparation of IgG

Normal IgG and ANCA-positive IgG were prepared from plasma of normal volunteers and patients with active MPO-ANCA- or PR3-ANCA-positive primary small vessel vasculitis, using a High-Trap-protein G column on an AKTA-FPLC system (GE Biosciences, South San Francisco, USA). None of these patients had dual positivity of PR3-ANCA and MPO-ANCA. Preparation of IgG was performed according to the methods described previously [17], [19]. We obtained written informed consent from all participants involved in our study. The research was in compliance of the Declaration of Helsinki and approved by the clinical research ethics committee of the Peking University First Hospital.

### Neutrophil isolation

Neutrophils were isolated from heparinized venous blood of healthy donors by density gradient centrifugation on Lymphoprep (Nycomed, Oslo, Norway). Erythrocytes were lysed with ice-cold ammonium chloride buffer, and neutrophils were washed in Hanks balanced salt solution without Ca^2+^/Mg ^2+^ (HBSS−/−; Chemical reagents, Beijing, China). Neutrophils were then suspended in HBSS with Ca^2+^/Mg^2+^(HBSS+/+; Chemical reagents, Beijing, China) to a concentration of 2.5×10^6^ cells/ml and used for PR3 and MPO membrane expression analysis, respiratory burst measurements, neutrophils degranulation and Western blot analysis [17].

### P38MAPK, ERK, JNK and PI3K inhibition

Flow cytometry was used to evaluate the effect of the p38MAPK inhibitor (SB202190) (Sigma-Aldrich, Louis, USA), the ERK inhibitor (PD98059) (Sigma-Aldrich, Louis, USA), the JNK inhibitor (6o) (Tocris, Louis, USA) and the PI3K inhibitor (LY294002) (Sigma-Aldrich, Louis, USA) on PR3 and MPO expression on neutrophils, as well as neutrophil respiratory burst, respectively. It was found by Manthey et al. that SB202190 blocked p38MAPK at 30 µM and did not inhibit ERK and JNK activity [20]. PD98059 was a highly selective inhibitor of ERK1 and ERK2 with the half maximal inhibitory concentration (IC50) of 4 µM and 50 µM respectively and did not inhibit activation of other highly related protein kinases [21]–[23]. 6o inhibited JNK1, JNK2 and JNK3 at 52 nM but did not block other kinases, including ERK2 and p38MAPK [24]. LY294002 at 50 µM specifically abolished PI3K activity but did not inhibit other protein kinases, including MAPK, protein kinase A, and protein kinase C [25]. The concentration dependence of the effect of all the inhibitors has been investigated in the above-mentioned studies [20]–[25]. Therefore, we selected SB202190 at 30 µM, PD98059 at 50 µM, 6o at 52 nM and LY294002 at 50 µM for the experiments. Toxicity of all the inhibitors to neutrophils had been examined by FACS using a Cell Apoptosis Detection Kit (BD Biosciences, California, USA). Pre-incubated with inhibitors, the proportion of living cells was higher than 90%. None of the inhibitors in such concentrations induced a potential cell apoptosis. Cells were pre-incubated with 30 µM SB202190, or 50 µM PD98059, or 50 µM LY294002, or the mixture of above-mentioned three inhibitors, or 52 nM 6o, or its vehicle, DMSO, as control, for 30 min, followed by other treatments.

### Measurement of respiratory burst by oxidation of dihydrorhodamine (DHR) to rhodamine

The generation of reactive oxygen radicals was assessed using DHR. This method was based on the fact that reactive oxygen radicals cause an oxidation of the nonfluorescence DHR to the green fluorescence rhodamine. Isolated neutrophils were gradually warmed to 37°C and incubated with 0.05 mM DHR123 (Sigma-Aldrich, Louis, USA) for 10 min at 37°C. Sodium azide (NaN_3_) (2 mM) was added in order to prevent intracellular breakdown of H_2_O_2_ by catalase. When indicated, cells were pre-incubated with the above specific inhibitors or its vehicle, DMSO, as control for 30 min at 37°C before the priming. Then, neutrophils were primed with 100 ng/ml C5a for 15 min at 37°C and incubated with patient-derived ANCA-positive IgG (200 µg/ml), normal IgG or monoclonal IgG1 antibody for 1 h at 37°C. The reaction was stopped by addition of 1 ml of ice-cold HBSS/1% BSA. Samples were kept on ice and analyzed using a FACScan. Neutrophils were identified in the scatter diagram, and data were collected from 10,000 cells per sample. The shift of green fluorescence in the FL-1 mode was determined. For each condition, the MFI (representing the amount of generated reactive oxygen radicals) was reported [17], [19].

**Figure 2:**
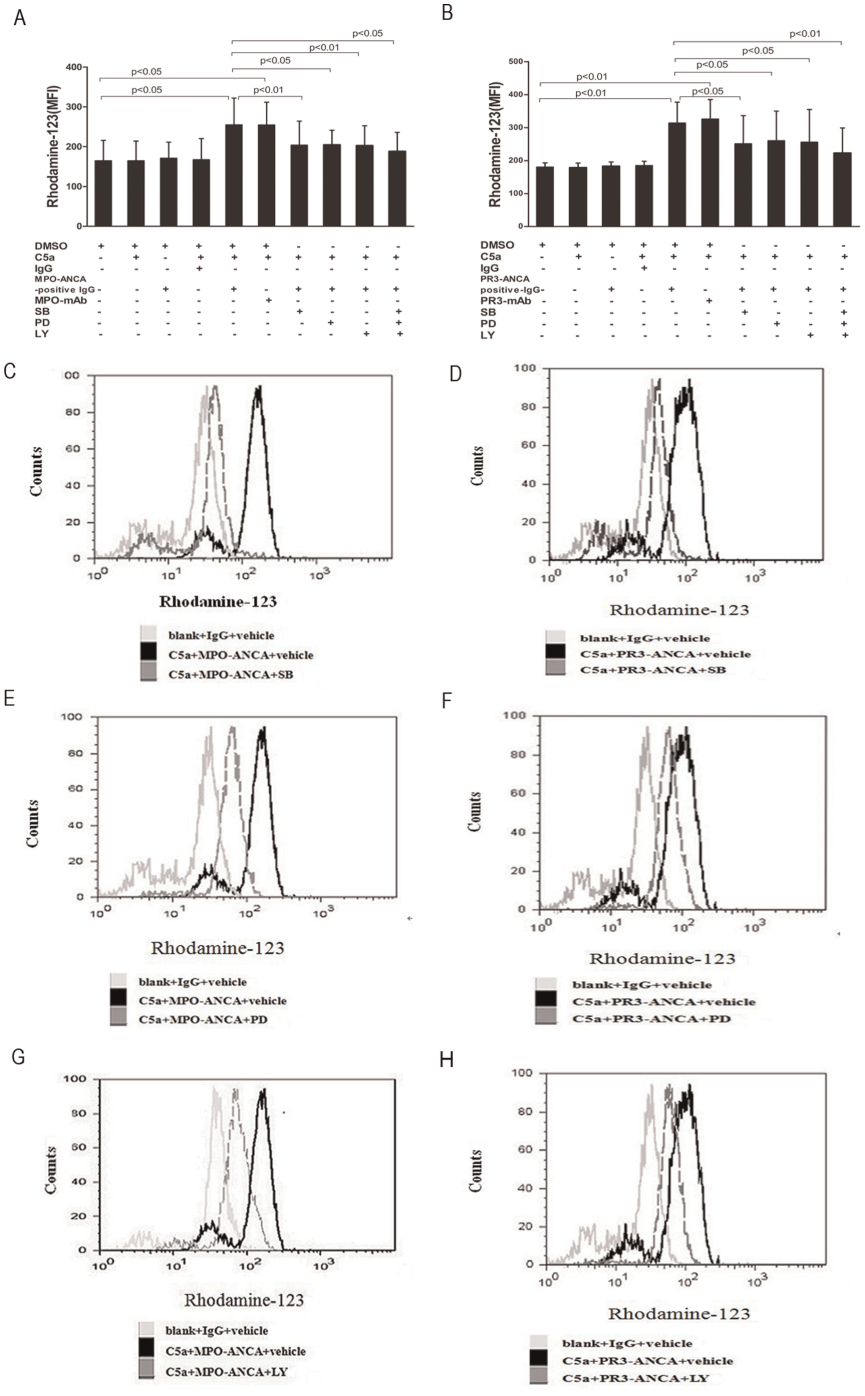
Neutrophil respiratory burst induced by patient-derived MPO-ANCA-positive IgG or PR3-ANCA-positive IgG in C5a-primed cells. Neutrophil respiratory burst induced by patient-derived MPO-ANCA-positive IgG (A) or PR3-ANCA-positive IgG (B) was measured by conversion of dihydrorhodamine-123 (DHR-123) to rhodamine-123 in C5a-primed cells in the presence and absence of SB202190, PD98059, LY294002 or the mixture of above-mentioned three inhibitors, respectively. Differences of MFI between groups were assessed using the paired t test. C–H were representative histograms showing that MPO-ANCA-positive IgG and PR3-ANCA-positive IgG induced respiratory burst in C5a-primed neutrophils and that inhibition of p38MAPK, ERK and PI3K reduced the ANCA-induced respiratory burst. Bars represent mean±SD of 5 MPO-ANCA-positive IgG and 3 PR3-ANCA-positive IgG preparations, each measured on neutrophils of 10 independent experiments and donors.

### Western blot analysis of phospho-p38MAPK, phospho-ERK, phospho-JNK and phospho-Akt

Neutrophils were stimulated with C5a 100 ng/ml or buffer for 15 min followed by stimulation with MPO-ANCA-positive IgG or PR3-ANCA-positive IgG, normal IgG or buffer control for 1 h, respectively. Samples were harvested and cell lysates were prepared by resuspending 1×10^6^ cells in 100 µl of ice-cold lysing solution (1∶100 phosphatase inhibitor). Samples were stored for 20 min on ice and centrifuged at 12,000 rpm for 5 min at 4°C. Supernatant was collected and protein concentration was measured. Samples were incubated for 5 min at 95°C in loading buffer (250 mM Tris-HCl [pH 6.8] with 4% sodium dodecyl sulfate, 20% glycerol, 0.01% bromphenol blue, 6% β-mercaptoethanol) and 50 µg of protein per lane were loaded on 10% sodium dodecyl sulfate-polyacrylamide gel, electrophoresed, and blotted onto nitrocellulose membrane by semidry equipment. Membrane was blocked in 5% BSA/0.05% Tween 20/tris-buffered saline (TBST) 1 h at room temperature. Phosphotyrosine was detected using specific monoclonal rabbit antibodies to phospho-p38MAPK, phospho-ERK, phospho-JNK and phospho-Akt (CST, Beverly, MA, USA), respectively (1∶1,000 dilution, 1∶2,000 dilution, 1∶1,000 dilution and 1∶2,000 dilution, respectively). Total protein was determined using specific monoclonal mouse antibodies to p38MAPK, ERK, JNK and Akt (CST, Beverly, MA, USA), respectively (1∶1,000 dilution, respectively). The nitrocellulose membrane was incubated overnight at 4°C with gentle agitation, followed by three washes with TBST for 10 min each time. The strips were then incubated with peroxidase-conjugated affinity-purified goat anti-mouse IgG or goat anti-rabbit IgG (Sigma-Aldrich, Louis, USA) diluted at 1∶10,000 with TBST/5%BSA for 1 h at room temperature with gentle agitation. Finally, they were revealed on autoradiographic film using ECL Plus Western Blotting Detection System (GE Healthcare, Piscataway, NJ). Protein levels were quantified using ImageJ software (National Institutes of Health, Bethesda, MD) [26].

### ANCAs activated C5a-primed neutrophils degranulation

Lactoferrin, an iron binding multifunctional glycoprotein that was an abundant component of the specific granules of neutrophils [27], [28], was considered as a biomarker of neutrophil degranulation [29], [30]. Neutrophils were stimulated with C5a 100 ng/ml or buffer for 15 min followed by stimulation with MPO-ANCA-positive IgG or PR3-ANCA-positive IgG, normal IgG or buffer control for 1 h, respectively. Cells were pre-incubated with the above specific inhibitors or its vehicle, DMSO, as control for 30 min at 37°C before the priming. Lactoferrin in the neutrophils supernatant, as a measure of neutrophil degranulation, were tested by ELISA using a commercial kit (USCNK, China). The ELISA procedure of measuring lactoferrin was as described previously [31]. In brief, the microtiter plate was pre-coated with a monoclonal antibody specific to lactoferrin. Supernatant of neutrophils degranulation reaction at dilutions of 1∶500 and standards were then added to the appropriate microtiter plate wells with a biotin-conjugated polyclonal antibody preparation specific for lactoferrin. Next, avidin conjugated horseradish peroxidase (HRP) was added to each microplate well and incubated. Then a tetramethylbenzidine (TMB) substrate solution was added to each well. Only those wells that contained lactoferrin, the biotin-conjugated antibodies and enzyme-conjugated avidin would exhibit a change in color. The enzyme-substrate reaction was terminated by the addition of a sulphuric acid solution and the color change was measured spectrophotometrically at a wave-length of 450 nm±10 nm. The concentrations of lactoferrin in the samples were then determined by comparing the OD value of the samples to the standard curve. The inhibition rate was calculated according to the following formula:

Inhibition rate =  (MFI_DMSO+C5a+MPO/PR3-ANCA_-MFI_SB/PD/LY+C5a+MPO/PR3-ANCA_)/

(MFI_DMSO+C5a+MPO/PR3-ANCA_ -MFI_DMSO_)×100%

### Membrane expression of PR3 and MPO on neutrophils after priming

Flow cytometry was used to evaluate PR3 and MPO expression on neutrophils. Cells were incubated with C5a (10, 100, 500 and 1000 ng/ml) (Biovision, San Francisco, USA) or buffer control for 15 min at 37°C. All further steps were performed on ice and washing steps were carried out using HBSS+/+containing 1% bovine serum albumin (BSA). Neutrophils were incubated with 0.5 mg/ml heat-aggregated goat IgG for 15 min to saturate Fcγ receptors. Next, cells were stained with a saturating dose of mouse monoclonal IgG1 antibody directed against human PR3 or MPO (Abcam, Cambridge, UK) or with an irrelevant IgG1 control antibody (Biolegend, California, USA) for 30 min. Neutrophils were then incubated with phycoerythrin (PE)-conjugated goat anti-mouse antibody (Abcam, Cambridge, UK) in the presence of 0.5 mg/ml heat-aggregated goat IgG. Fluorescence intensity of PE was analyzed using flow cytometry assessment of ANCA-antigen expression. Samples were analyzed using a FACScan (Becton Dickinson, Germany). Neutrophils were identified in the scatter diagram, and data were collected from 10,000 cells per sample. The level of PR3- or MPO-expression was calculated as MFI of specific binding of the isotype control antibody. For the inhibition test, cells were pre-incubated with 30 µM SB202190, or 50 µM PD98059, or 50 µM LY294002, or the mixture of above-mentioned three inhibitors, or 52 nM 6o, or its vehicle, as control, for 30 min on ice, followed by other treatments [17].

**Figure 3:**
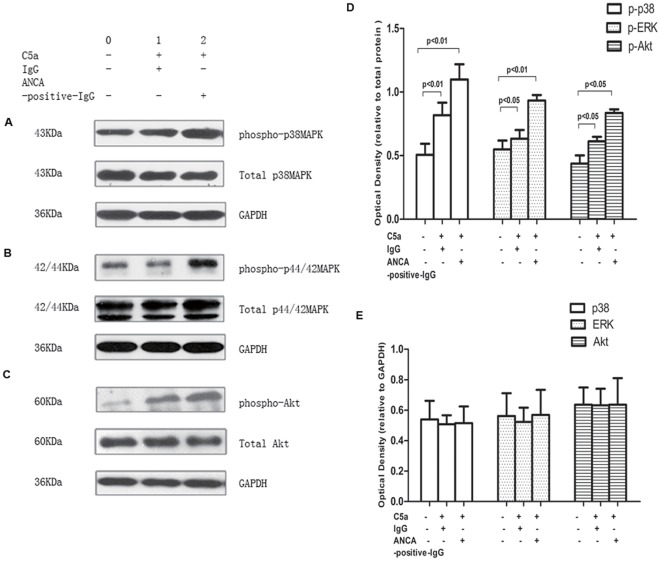
Western blot analysis for phospho-p38MAPK, phospho-ERK and phospho-Akt. Neutrophils were stimulated with C5a 100 ng/ml or buffer for 15 min followed by stimulation with normal IgG, MPO-ANCA-positive IgG or PR3-ANCA-positive IgG, and buffer control, respectively. Samples were harvested and phospho-p38MAPK (pp38, A), phospho-ERK (pERK, B) and phospho-Akt (pAkt, C), were determined by immunoblotting. A representative example of 4 independent experiments is shown. GAPDH is shown as loading control. 0: blank, 1: C5a+isotype control; 2: C5a+ANCA-positive IgG. The corresponding densitometric analysis was shown in D for phosphorylated protein relative to total protein and E for total protein relative to GAPDH (n = 4). Differences of optical density between groups were assessed using the t test.

**Figure 4:**
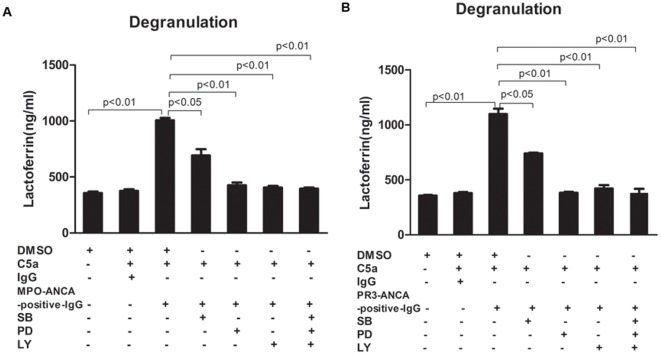
P38MAPK, ERK and PI3K pathway inhibitors block C5a-primed neutrophils for ANCA-induced degranulation. ANCA-induced neutrophil degranulation was determined by measuring the lactoferrin concentrations in the supernatant of neutrophil degranulation reaction. Inhibition of p38MAPK, ERK and PI3K reduced ANCA-induced lactoferrin release. Bars represent mean±SD of 3 MPO-ANCA and 3 PR3-ANCA preparations, measured on neutrophils of 4 donors. T test was used to compare concentration of lactoferrin between groups.

### Statistical analysis

Shapiro-Wilk test was used to examine whether the data was normally distributed. Quantitative data were expressed as means±SD (for data that were normally distributed) or median and range (for data that were not normally distributed). Differences of quantitative parameters between groups were assessed using the t test (for data that were normally distributed) or Mann-Whitney U test (for data that were not normally distributed) as appropriate. Differences were considered significant when *P*<0.05. Analysis was performed with SPSS statistical software package (version 13.0, Chicago, IL, USA).

## Results

### C5a increased expression of membrane-bound PR3 (mPR3) on neutrophils

Expression of mPR3 on neutrophils of 11 healthy donors was analyzed. Neutrophils were incubated with different concentrations of C5a (10, 100, 500 and 1000 ng/ml), and mPR3 expression was determined by flow cytometry. The level of mPR3 expression on neutrophils increased dose-dependently (mPR3 expression on neutrophils were 986.9±220.4, 1001.1±202.1, 1169±186.9, 1213±187.5, 1335.2±217.63, for 0, 10, 100, 500 and 1000 ng/ml C5a, respectively, expressed as MFI). Comparing with non-primed neutrophils, the level of mPR3 expression was significantly higher on neutrophils primed with C5a at concentrations of 100, 500 and 1000 ng/ml (*P*<0.001, t-test; *P*<0.001, t-test; *P*<0.001, t-test), respectively. The level of membrane-bound MPO (mMPO) expression on neutrophils were 993.3±91.3, 1055.5±178.8, 1060.3±172, 1062.2±201.7, 1063.5±200.1, for 0, 10, 100, 500 and 1000 ng/ml C5a, respectively, (p>0.05, t-test, compared with non-primed neutrophils, Figure 1). Increases in membrane-bound PR3 expression were much stronger during neutrophils priming compared with mMPO.

### P38MAPK, ERK and PI3K pathway inhibitors blocked C5a-primed neutrophils for ANCA-induced respiratory burst

We studied whether C5a primed neutrophils for ANCA-induced respiratory burst. ANCAs-postive IgG were prepared from 5 patients with active MPO-ANCA-positive vasculitis and 3 patients with active PR3-ANCA-positive vasculitis, respectively. Based on the observation described above that C5a at a concentration of 100 ng/ml significantly increased mPR3 expression on neutrophils, this concentration of C5a was employed for testing ANCA-induced respiratory burst. Compared with non-primed neutrophils, the MFI value increased significantly in C5a-primed neutrophils activated with PR3-ANCA-positive IgG and MPO-ANCA-positive IgG (314±63.1 vs. 180.2±12.9, *P*<0.01; 254.8±67.1 vs. 164.4±51.3, *P*<0.05, t-test, respectively) (Figure 2). No obvious respiratory burst activity was observed with C5a or ANCA-positive IgG alone (Figure 2). We next investigated whether C5a-primed neutrophils for ANCA-induced respiratory burst were dependent on activation of the p38MAPK, ERK, JNK and PI3K pathways. Neutrophils were pre-incubated with the above signal transduction inhibitors before the priming with C5a and the subsequent stimulation with ANCA. We used mAb to MPO or PR3 instead of human ANCA-positive IgG preparations for comparison. Pre-incubation of neutrophils with the p38MAPK inhibitor (SB202190), ERK inhibitor (PD98059), PI3K inhibitor (LY294002), and the mixture of above-mentioned three inhibitors decreased oxygen radical production in C5a primed neutrophils induced by ANCA-positive IgG from patients. Pre-incubation of neutrophils with the JNK inhibitor (6o) did not decrease oxygen radical production in C5a primed neutrophils induced by ANCA-positive IgG from patients (data not shown). In C5a-primed neutrophils, subsequently activating with MPO-ANCA-positive IgG, the MFI value was 254.8±67.1, which decreased to 203.6±60.3, 204.4±36.7, 202.4±49.9 and 188±47.9 upon pre-incubation with SB202190, PD98059, LY294002 and the mixture of above-mentioned three inhibitors (compared with that without inhibitors, *P*<0.01, *P*<0.05, *P<0.01* and *P*<0.05, t-test), respectively. For PR3-ANCA-positive IgG, the MFI value was 314±63.1 in C5a-primed neutrophils, which decreased to 251±85.2, 260.2±89.9, 255.8±98.9 and 222.6±76.3 upon pre-incubation with SB202190, PD98059, LY294002 and the mixture of above-mentioned three inhibitors (compared with that without inhibitors, *P*<0.05, *P*<0.05, *P<0.05* and *P*<0.01, t-test), respectively (Figure 2).

### Western blot analysis for phospho-p38MAPK, phospho-ERK, phospho-JNK and phospho-Akt in C5a-primed neutrophils activated by ANCA

Western blot analysis was performed to study tyrosine phosphorylation of p38MAPK, ERK, JNK and Akt, respectively. Using monoclonal antibodies that detect the phosphorylated forms of each kinase, we analyzed the effect of C5a priming as well as the effect of the subsequent MPO-ANCA-positive IgG or PR3-ANCA-positive IgG, stimulation. Figure 3 showed a representative Western blot analysis of phosphorylated p38MAPK, phosphorylated ERK, and phosphorylated Akt and the corresponding Western blot analysis of total p38MAPK, ERK and Akt. We observed significantly increased phosphorylation kinases by pre-incubation with C5a or C5a plus ANCA-positive IgG. Neither increased phosphorylation nor increased total JNK in C5a primed neutrophils induced by PR3-ANCA-positive IgG or MPO-ANCA-positive IgG was observed (data not shown). There are no significant changes of total kinase by preincubation with C5a and ANCA-positive IgG at the mRNA level (see the File S1, Table S1 and Figure S1). These data suggested an important role for the p38MAPK, ERK and PI3K pathways in C5a-mediated priming of neutrophils.

**Figure 5:**
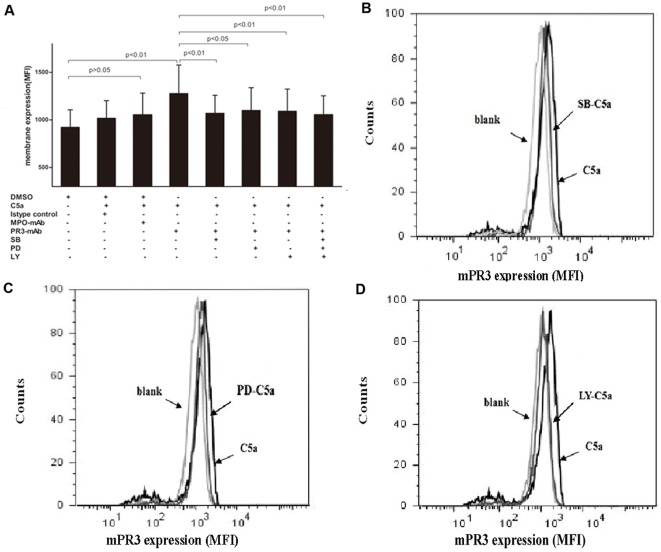
Effects of the p38MAPK inhibitor, ERK and PI3K inhibitor on translocation of PR3. A: Neutrophils were incubated with the SB202190, PD98059, LY294002 or the inhibitors mixture or vehicle for 30 min prior to incubation with C5a (100 ng/ml). Bars represent mean±SD of repeated measurements on neutrophils of 10 independent experiments and donors. T test was used for comparison. B-D: a representative histogram of effects of the above inhibitors on translocation of PR3 upon C5a priming.

### P38MAPK, ERK and PI3K pathway inhibitors blocked C5a-primed neutrophils for ANCA-induced degranulation

ANCA-induced neutrophil degranulation was determined by measuring the lactoferrin concentration in the supernatant. Pretreatment with p38MAPK, ERK, PI3K inhibitors or the mixture of above-mentioned three inhibitors reduced PR3-ANCA-positive IgG-induced and MPO-ANCA-positive IgG-induced lactoferrin release. The lactoferrin concentration increased from 356.9±23.9 ng/ml in the non-primed neutrophils supernatant to 1099.8±80.7 ng/ml in C5a-primed neutrophils induced by PR3-ANCA-positive IgG supernatant (*P*<0.01, t-test), and decreased to 739.3±18.5 ng/ml, 383.3±20.4 ng/ml, 422.1±52.5 ng/ml and 378±69.3 ng/ml upon pre-incubation with SB202190, PD98059, LY294002 and the mixture of above-mentioned three inhibitors (compared with that without inhibitors, *P*<0.05, *P*<0.01, *P*<0.01, and *P*<0.01, t-test, the inhibition rate was 48.02±8.79%, 96.47±1.84%, 91.48±4.62% and 97.21±5.97%), respectively. In C5a-primed neutrophils induced by MPO-ANCA-positive IgG, the lactoferrin concentration in the supernatant increased from 359.9±23.9 ng/ml in untreated cells to 1007.4±34.9 ng/ml (*P*<0.01, t-test), which decreased to 691.7±98.5 ng/ml, 427.0±40.2 ng/ml, 405.5±25.6 ng/ml and 395.7±16.9 ng/ml upon pre-incubation with SB202190, PD98059, LY294002 and the mixture of above-mentioned three inhibitors (compared with that without inhibitors, *P*<0.05, *P*<0.01, *P*<0.01 and *P*<0.01, t-test, the inhibition rate was 49.40±14.74%, 89.00±4.01%, 92.92±7.32% and 94.35±5.95%), respectively (Figure 4). The inhibition rate of PI3K inhibitor was significantly higher than that of p38MAPK inhibitor in PR3-ANCA-positive IgG and MPO-ANCA-positive IgG mediated neutrophils degranulation (*P*<0.05 and *P*<0.05, t-test, respectively). The inhibition rate of ERK inhibitor was significantly higher than that of p38MAPK inhibitor in PR3-ANCA-mediated neutrophils degranulation (*P*<0.05, t-test). The inhibition rate of ERK inhibitor tended to be significantly higher than that of p38MAPK inhibitor in MPO-ANCA-mediated neutrophils degranulation (*P* = 0.066, t-test). Pretreatment with JNK inhibitor did not reduce PR3-ANCA-positive IgG-induced and MPO-ANCA-positive IgG-induced lactoferrin release (data not shown).

### Effects of the p38MAPK, ERK, JNK and PI3K inhibitor on translocation of PR3

We studied a possible mechanism by which the p38MAPK, ERK, JNK and PI3K pathways might control ANCA-stimulated respiratory burst in C5a-primed neutrophils. Since we previously found increases in mPR3 expression are much stronger during neutrophils priming compared with MPO, we only explored whether p38MAPK, ERK, JNK or PI3K pathway controlled the C5a-mediated translocation of PR3 to the cell surface. Using flow cytometry, we showed parallel experiments that inhibiting signal pathway with SB202190, PD98059, LY294002 and the mixture of above-mentioned three inhibitors resulted in a decreased C5a-induced translocation of PR3. mPR3 expression increased from 923.3±182.4 in untreated cells to 1278.3±299.3 after C5a treatment and decreased to 1069.9±188.9, 1100±238.2, 1092.3±231.8 and 1053.9±200.3 by SB202190, PD98059, LY294002 and the mixture of above-mentioned three inhibitors (compared with that without inhibitors, *P*<0.01, *P*<0.05, *P*<0.01 and *P*<0.01, t-test), respectively (Figure 5). Pretreatment with JNK inhibitor did not reduce C5a-mediated translocation of PR3 to the cell surface (data not shown). Together, these experiments indicated that p38MAPK, ERK and PI3K pathways controlled the C5a-mediated translocation of PR3 from the intracellular granules to the cell surface.

## Discussion

ANCA-induced neutrophils respiratory burst is an important contributor to the development of ANCA-associated vasculitis. Recent studies, both in the mouse model and in human, suggested that complement activation is involved in the pathogenesis of AAV [9]–[11]. Among the complement activation products, C5a is one of the most potent inflammatory peptide, with a broad spectrum of functions. C5a is a strong chemoattractant for neutrophils and also has chemotactic activity for monocytes and macrophages (reviewed by Guo *et al.* [32]). C5a exerts its effects through the high-affinity C5a receptor. Recent investigations by Schreiber et al. demonstrated that ANCA-stimulated neutrophils activate complement and generate C5a. In turn, C5a was found to prime neutrophils dose-dependently for ANCA-induced respiratory burst [12], indicating a pivotal role of C5a and its receptor on neutrophils in disease induction. The current study confirms and extends these observations. Schreiber et al. reported that C5a-conditioned serum could increase mPR3 expression on neutrophils. Our study confirmed this observation by demonstrating that purified recombinant C5a dose-dependently increased neutrophil mPR3 expression. Interestingly however, both studies demonstrated that after incubation with C5a (or C5a-conditioned serum), increased in membrane-bound MPO expression are much lower than membrane-bound PR3 expression. This result was in line with some other studies [12], [33]–[35]. Witko-Sarsat V et al [36] found that MPO was mainly released into the extracellular medium and PR3 was released in minute amounts into the extracellular medium, providing additional evidence that MPO mobilization is different from that of PR3. In the current study, the concentrations of secreted MPO were measured in the supernatant of C5a-stimulated neutrophils using specific ELISA Kit. It was found that the concentration of extracellular release of MPO from neutrophils with C5a-priming was significantly higher than that without C5a-priming (see the File S1).

Several signal transduction studies have been performed to better understand how ANCAs activated neutrophils [15], [16], [37]. The most important finding in the current study was that p38MAPK, ERK and PI3K inhibition blocked C5a-dependent ANCA-mediated neutrophil activation and degranulation; p38MAPK, ERK and PI3K inhibitors blocked C5a-primed neutrophils for ANCA-induced respiratory burst via inhibition of ANCA target antigen translocation. These results could explain, at least partially, the observation by van der Veen et al. that p38MAPK inhibition had only a moderate beneficial effect on disease severity in the mouse model of anti-MPO IgG/LPS-induced glomerulonephritis [16].

Our results suggested that C5a-mediated neutrophil activation were different from that TNFα-mediated. It was found by Kettritz et al. that p38MAPK controls the translocation of ANCA antigens to the cell surface in the TNFα-mediated priming process; however, ERK and PI3K were not involved in this translocation [15]. In multiple organ dysfunction syndrome (MODS), it was found that p38MAPK, not ERK, played a major role in the C5a enhancement of lipopolysaccharide (LPS)-induced interleukin-6 (IL-6) and TNF-α production in peripheral blood mononuclear cells (PBMCs) [26]. The important precondition for C5a-triggered neutrophil activation by ANCA is the accessibility of ANCA antigens on the cell surface, signaling mechanisms involved in priming by C5a is necessary. The interactions between C5a receptor and related proteins on the surface of neutrophils would determine the signal-transduction of PR3 membrane expression. However, the C5a receptor and TNFα receptor activated different downstream signaling molecules subsequently.

P38MAPK, ERK and PI3K inhibitors mixture blocked C5a-primed neutrophils for ANCA-induced respiratory burst to some extent but not completely. According to these results, we speculated that there were other pathways involved in this process.

Utilizing small interfering RNAs to silence kinase-associated genes holds promise in the study of the signal pathways strategy. However, because of the difficulty to cultivate neutrophils which were extracted from peripheral blood and the well-known short circulatory half-life of neutrophils, it is technically difficult to use small interfering RNAs technique to knockdown specific signaling pathway in neutrophils. This is a limitation of the experiment system in the current study.

In conclusion, activation of p38MAPK, ERK and PI3K were all important steps in the translocation of ANCA antigens and activation of neutrophils by ANCA. Inhibiting each of these pathways resulted in decreased respiratory burst by C5a-mediated priming. Each kinase controlled the translocation of ANCA antigens to the cell surface. Pharmacologic blockade of p38MAPK, ERK and PI3K might limit inflammatory damage caused by ANCA-activated neutrophils.

## Supporting Information

File S1 **mRNA expression of p38MAPK, ERK1/2 and PI3K in C5a-induced ANCA mediated neutrophils activation and the MPO Concentration in C5a-primed neutrophils supernatant.**
(DOC)

Table S1 **Primers used in Q-RT-PCR.**
(DOC)

Figure S1 Q-RT-PCR analysis of mRNA p38MAPK, ERK1/2 and PI3K expression in total RNA extracts from control and C5a-induced ANCA mediated neutrophils. Data of 2^−ΔΔC^_T_ were expressed by means ± SD (n = 3). Differences of fold change between groups were assessed using the t test.
ΔΔC_T_ = (C_ T p38MAPK/ERK/PI3K_-C _T GAPDH_) _C5a+ANCA-positive-IgG_ -(C _T p38MAPK/ERK/PI3K_-C _T_
(TIF)
