# Supplementary material for: p38MAPK, ERK and PI3K Signaling Pathways Are Involved in C5a-Primed Neutrophils for ANCA-Mediated Activation
Source: PLoS One. 2012 May 31;7(5):e38317. doi: 10.1371/journal.pone.0038317 (PMC3365028; doi:10.1371/journal.pone.0038317)
Supplement: File S1 — mRNA expression of p38MAPK, ERK1/2 and PI3K in C5a-induced ANCA mediated neutrophils activation and the MPO Concentration in C5a-primed neutrophils supernatant. (DOC) [file pone.0038317.s001.doc]

**File S1**

**Methods**

**Quantitative RT-PCR analysis**

RNA was extracted using Trizol extraction liquid (invitrogen, California, USA). Quantitative RT-PCR (Q-RT-PCR) was performed using GoTaq ® 2-Step RT-qPCR System kit (Promega, Madison, USA). GAPDH mRNA was used as internal controls. RNA tested and primer sequences were listed in Table S1. mRNA p38MAPK, ERK1/2 and PI3K expression in total RNA extracts from control and C5a-induced ANCA-mediated neutrophils was analysed by Q-RT-PCR. The Q-RT-PCR procedure was as described previously [1]. Data were expressed as means ± SD of 2^-ΔΔC^_T_ (n=3).

ΔΔC_T_=(C _T p38MAPK/ERK/PI3K_-C _T GAPDH_) _C5a+ANCA-positive-IgG_ -(C _T p38MAPK/ERK/PI3K_-C _T GAPDH_)_control_

**ELISA**

Myeloperoxidase (MPO) in the C5a-primed neutrophils supernatant were tested by ELISA using a commercial kit (USCNK, China). The ELISA procedure of measuring MPO was as described previously [2]. In brief, the microtiter plate provided in this kit has been pre-coated with an antibody specific to MPO. Supernatant of neutrophils at dilutions of 1:200 and standards were then added to the appropriate microtiter plate wells with a biotin-conjugated antibody preparation specific for MPO. Next, Avidin conjugated to Horseradish Peroxidase (HRP) was added to each microplate well and incubated. After TMB substrate solution was added, only those wells that contain MPO, biotin-conjugated antibody and enzyme-conjugated Avidin would exhibit a change in color. The enzyme-substrate reaction was terminated by the addition of sulphuric acid solution and the color change is measured spectrophotometrically at a wavelength of 450nm. The concentration of MPO in the samples was then determined by comparing the O.D. of the samples to the standard curve.

**Results**

**mRNA expression of p38MAPK, ERK1/2 and PI3K in C5a-induced ANCA mediated neutrophils activation**

The result of the mRNA expression of p38MAPK, ERK1/2 and PI3K was shown in Figure S1. No significant differences were found between the control and the groups pretreated with C5a and ANCA treatment (P>0.05, t-test).

**The MPO Concentration increased in C5a-primed neutrophils supernatant**

MPO was detected at the C5a-primed neutrophils membrane but may be mainly released into the extracellular medium. The MPO concentration increased from 1055±124.9 ng/ml in the non-primed neutrophils supernatant to 1248.2±127.3 ng/ml in C5a-primed neutrophils supernatant (*P*<0.05, t-test).

**References**

1. Blaise S, Kneib M, Rousseau A, Gambino F, Chenard MP, et al. (2012) In vivo evidence that RAF4 is required for central nervous system myelin homeostasis. PLoS One 7: e30917.

2. Witko-Sarsat V, Cramer EM, Hieblot C, Guichard J, Nusbaum P, et al. (1999) Presence of proteinase 3 in secretory vesicles: evidence of a novel, highly mobilizable intracellular pool distinct from azurophil granules. Blood 94: 2487-249.
