# Supplementary material for: p38MAPK, ERK and PI3K Signaling Pathways Are Involved in C5a-Primed Neutrophils for ANCA-Mediated Activation
Source: PLoS One. 2012 May 31;7(5):e38317. doi: 10.1371/journal.pone.0038317 (PMC3365028; doi:10.1371/journal.pone.0038317)
Supplement: Table S1 — Primers used in Q-RT-PCR. (DOC) [file pone.0038317.s002.doc]

**Table S1. Primers used in Q-RT-PCR**

| mRNA | Sense probe (5’-3’) | Antisense probe (5’-3’) |
| --- | --- | --- |
| GAPDH | AGAAGGCTGGGGCTCATTTG | AGGGGCCATCCACAGTCTTC |
| P38MAPK | GAGCGTTACCAGAACCTGTCTC | CGTAGCCTGTCATTTCATCATC |
| ERK1/2 | GGCTTCCTGACGGAGTATGT | TCTTGGAGGGCAGAGACTGTA |
| PI3K | CAGGGCTTTCTGTCTCCTCTAA | TGATGTAGTGTGTGGCTGTTGA |
