# Supplementary figures and images for: p38MAPK, ERK and PI3K Signaling Pathways Are Involved in C5a-Primed Neutrophils for ANCA-Mediated Activation
Source: PLoS One. 2012 May 31;7(5):e38317. doi: 10.1371/journal.pone.0038317 (PMC3365028; doi:10.1371/journal.pone.0038317)

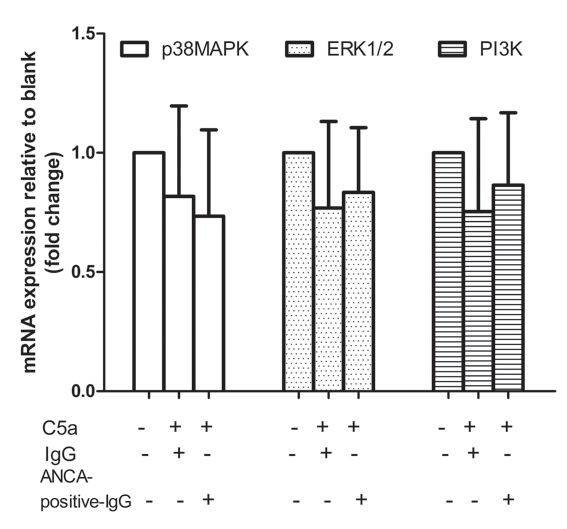

Supplement: Figure S1 — Q-RT-PCR analysis of mRNA p38MAPK, ERK1/2 and PI3K expression in total RNA extracts from control and C5a-induced ANCA mediated neutrophils. Data of 2−ΔΔC T were expressed by means ± SD (n = 3). Differences of fold change between groups were assessed using the t test. ΔΔCT = (C T p38MAPK/ERK/PI3K-C T GAPDH) C5a+ANCA-positive-IgG -(C T p38MAPK/ERK/PI3K-C T (TIF) [file pone.0038317.s003.tif]
